# China declared malaria-free: a milestone in the world malaria eradication and Chinese public health

**DOI:** 10.1186/s40249-021-00882-9

**Published:** 2021-07-12

**Authors:** Xiao-Nong Zhou

**Affiliations:** 1 National Institute of Parasitic Diseases at the Chinese Centre for Disease Control and Prevention & Chinese Centre for Tropical Diseases Research, Shanghai, 200025 People’s Republic of China; 2 School of Global Health, Chinese Centre for Tropical Diseases Research, Shanghai Jiao Tong University School of Medicine, Shanghai, 200025 People’s Republic of China

On 30 June 2021, the World Health Organization announced that the People’s Republic of China (PR China) had been certified malaria-free [1], after a field mission in PR China in May 2021 by an independent certification panel. This award of malaria-free certification is a major milestone both for the world history in malaria eradication programme and for the Chinese public health history. Malaria is one of the top three infectious diseases in terms of disease burden and impacts on the world population. According to the World Malaria Report, 229 million people had malaria and 409 000 people died of it in 2019 [2]. Malaria has a long history in China. The Chinese word of malaria was found on the oracle bone and bronze inscriptions of the Shang Yin era from 1562 BC to 1066 BC, which indicated malaria had prevailed for more than 3000 years in China. Malaria prevailed in 24 provinces/municipalities/autonomous regions and was one of the most severe infectious diseases in the country. The infection affected the health status of local people with massive burden of disease. For example, 30 million malaria cases occurred with more than 1% prevalence before 1949 [3].

After the founding of the PR China in 1949, an important agenda for the control of infectious diseases, including malaria, was set up by the Central Government of China. A total of four phases in the national malaria control programme were carried out. These include: focal investigation and control phase (1949–1959); severe epidemic phase (1960–1979); incidence rate reduction phase (1980–1999); and consolidation phase (2000–2009). Following a 60-year effort of the national malaria control programme, transmission of *Plasmodium falciparum* was interrupted in all provinces except Yunnan Province and malaria incidence further reduced in other areas. Moreover, no malaria cases had been reported for four consecutive years in 1687 out of 2858 (59.03%) malaria-endemic counties, cities and districts in the country by 2009. In addition, the incidence rate was less than 10/10 000 in all counties of the country, except only in four counties where malaria incidence rate was more than 10/10 000. These achievements paved the way to initiate the national malaria elimination programme in 2010. Thereafter, after another 10 year’s effort, both the number of malaria cases and the distribution of malaria endemic areas were reduced significantly. The last indigenous malaria case was reported in April 2016 in Yunnan Province, and zero indigenous case has been since 2017 [4].

How did China come to a malaria-free country in only seven decades? This is a question concerned by most of the malaria experts as well as experts on public health. The primary lessons learnt from the national control programme in 1950–2009 have been summarized in the following aspects: (i) high commitment by government at all levels with coordinating resources, including financial and human resources to be sustainably invested in national malaria control programmes; (ii) robust working mechanism to promote the actions on multi-sectoral, multi-regional and multi-disciplinary cooperation, which ensure the activities performed in time; (iii) innovative researches produced rich tools used in the national malaria control programmes, including updating the strategies in different control phases and developing new chemotherapy drugs and schemes, diagnostics, surveillance kits and schemes, insecticide-treated bed-nets, and ways for information-education-communication; (iv) deep integration between western medicine and Chinese traditional medicine allowed the Chinese Nobel Prize winner Tu Youyou to discover artemisinin which contributed significantly to saving life of world population; (v) active communities involvement to modify mosquito habitats and improve sanitation through patriotic public health campaign; (vi) international cooperation and communication to ensure all steps in control activities along with international standards.

With regard to the national malaria elimination programme initiated in 2010, the main activities related to the malaria elimination were summarized as follows: (i) the *Action Plan for Malaria Elimination in China* (2010–2020) was issued by the Chinese Ministry of Health together with other 12 Ministries of the Central Government in 2010; (ii) stratified strategy with four catalogues of epidemic status was implemented to strengthen malaria surveillance and response; (iii) the “*National Information Management System for Parasitic Disease Control and Prevention*” was established in 2011 to improve the surveillance and response system during elimination phase; (iv) the strategy of "tracking cases, disposing of foci to eliminate infectious source" was formulated alongside with "1-3-7" norm which accelerated interruption of malaria transmission [5]; (v) the network of malaria diagnostic reference laboratories has been established in 2016 and covered all the 24 malaria-endemic provinces/municipalities/autonomous regions to improve quality control of malaria detection as well as case review and confirmation. (vi) the socio-economic improvement and implementation of poverty alleviation projects in remote and poverty-stricken mountain areas also contribute to and speed up malaria elimination. Following these efforts, no indigenous cases had been reported since 2017, marking no occurrence of indigenous malaria case for the first time in the history of the PR China, and the status of zero indigenous malaria case has been maintained up to date.

Malaria elimination is not the final goal for the public health of the PR China, rather, it is a new start point for China to promote work both on preventing imported malaria cases and on engaging global health to transfer Chinese approaches to other countries where malaria is still endemic. We expect that achievements from these approaches used in the Chinese national malaria control and elimination programme could contribute further to a healthy world to achieve the final goal of malaria eradication in the world.

## Data Availability

Not applicable.
